# Quantitative Characterization of Binding Pockets and Binding Complementarity by Means of Zernike Descriptors

**DOI:** 10.1021/acs.jcim.9b01066

**Published:** 2020-02-12

**Authors:** Lorenzo Di Rienzo, Edoardo Milanetti, Josephine Alba, Marco D’Abramo

**Affiliations:** † Department of Physics, Sapienza University of Rome, Piazzale Aldo Moro, 5, 00185 Rome, Italy; ‡ Center for Life Nano Science@Sapienza, Italian Institute of Technology, Viale Regina Elena 291, 00161 Rome, Italy; ¶ Department of Chemistry, Sapienza University of Rome, Piazzale Aldo Moro, 5, 00185 Rome, Italy

## Abstract

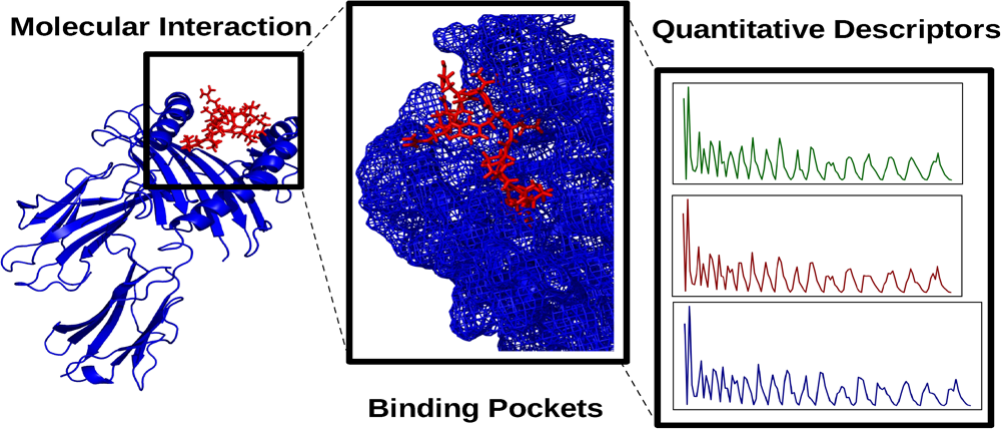

In this work, we describe the application of the Zernike formalism to quantitatively characterize the binding pockets of two sets of biologically relevant systems. Such an approach, when applied to molecular dynamics trajectories, is able to pinpoint the subtle differences between very similar molecular regions and their impact on the local propensity to ligand binding, allowing us to quantify such differences. The statistical robustness of our procedure suggests that it is very suitable to describe protein binding sites and protein–ligand interactions within a rigorous and well-defined framework.

## Introduction

Interactions between proteins and small molecules regulate many fundamental biological processes, and hence, their detailed description constitutes the basis for an efficient and rational drug design.^1^ In particular, the analysis of the chemical–physical properties of the binding site, as well as structural properties such as geometry, shape, and size, represents a crucial step to elucidate protein structure-based ligand design.^2,3^

Protein–ligand interaction typically occurs in a protein cavity, a specific region that is often not accessible to the bulk solvent, although it can be hydrated or free from any solvent molecule.^1^ Given a specific ligand, the identification of a protein region characterized by favorable interactions with it depends essentially on two factors.^3,4^ On one side, it is essential to take into account the shape complementarity between the binding site and the small molecule, in line with the key-lock paradigm.^5^ On the other side, the comparison of the physicochemical properties of the protein binding site and the ligand represents an additional information layer for pocket detection, which can be used to improve the estimation of the ligand binding affinity and specificity.^1^

Furthermore, it is well known that the connection between the structure and the function of a protein also relies on its conformational dynamics.^6−8^ In this respect, molecular dynamics (MD) simulations are a powerful tool that is able to describe the structural–dynamical behavior of the system at the atomic level of detail.^9,10^ Therefore, using MD-based approaches, it is possible to analyze the time evolution of a cavity by measuring its structural features, such as volume, solvent accessibility, and geometry changes.

In the past, several algorithms have been proposed to detect and describe protein cavities. According to the approach used, they can be mainly grouped into three different classes:^11−13^ The first group is based on geometrical or shape characterization,^2,14−18^ while the second group is composed by energy-based methods, which estimate probe-pocket interaction energy.^19−22^ The last group is formed by sequence-based methods exploiting the propensity of conserved residues belonging to the binding site.^23^

Although a plethora of cavity descriptors have been proposed, it is not easy to find a set of them that is able to univocally describe the feature of the region of interest on a more chemical–physical ground; that is, it is not easy to find a set of them that is independent from the choice and combination of specific descriptors. In fact, many of the methods developed to identify and characterize binding sites are based on a multiplicity of factors that are jointly considered to define a single score for each residue, which represents its binding propensity. Typically, the most used descriptors to characterize a binding site are the site size, the donor/acceptor character, the hydrophobic/hydrophilic properties, the measure of how the average site point interacts with the receptor via van der Waals nonbonded interactions with a specific probe, and the solvent exposure/accessibility. The binding site characterization therefore is usually linked to both the weight of each descriptor and how these weights are combined.^2,14,17,24−26^

Therefore, the interest in alternative methods that are able to characterize protein substrate recognition on a quantitative and rational ground is very high. In addition, a useful descriptor should be also able to give information on—and possibly, to predict—the interactions between the cavity and the interacting molecules.^27,28^ Here, we present a new computational protocol that is able to quantitatively describe both the shape and electrostatic properties of a given subregion of a protein on a rigorous ground by means of a moment-based approach using the Zernike polynomials.^29−31^

The main advantage of this method is its ability to describe subregions of a molecular surface in a compact way using a single vector of numbers to provide quantification of both the geometrical shape and the electrostatic potential. This characterization does not depend on any other measure and does not need any arbitrary choice, thus representing a simple description—still quantitative—of the chemico-physical characteristics of a molecular subregion.

The mathematical properties of the moment-based formalisms, and in particular, of the Zernike descriptors, allow absolute characterization of the selected region of the molecule, independent from the relative geometrical orientations of the protein or the ligand. Indeed, the approaches based on the Zernike moments provide a superposition-free description, which is invariant under rotation and translation.^32^ In particular, the method furnishes an ordered set of numbers that describes the geometrical shape and the electrostatic properties of the selected molecular surface, thus allowing us to easily compare cavities formed by different numbers of atoms.^33^

The selected region is thus described by three vectors of numbers: the first describes the shape of the given patch; the second and third are associated with positive and negative electrostatic potentials, respectively. These vectors can be then used to strikingly compare, for example, different pockets, a pocket and a ligand, and the time evolution of a pocket. Such an approach is applied here to (i) characterize the pocket conformational changes along an MD trajectory and (ii) evaluate the binding complementarity between a binding pocket and its cognate ligand.

The characterization of the pocket evolution as described by the Zernike polynomials has been applied to two members of Src family protein kinases (SFKs), namely, c-Src and Lck. The SKF proteins live at least in two conformations: the closed or inactive conformation and the open or active conformation.^34^ In the open conformation, the active site—the kinase domain (KD)—is more accessible to the substrate. Although c-Src and Lck have a KD sequence identity of 50%, they interact with different targets. In fact, c-Src is present in almost all cells, and it is able to phosphorylate a wide number of intracellular proteins. On the contrary, Lck is present only in T lymphocytes, and it is specific to phosphorylate the immune-receptor tyrosine-based activation motif (ITAM), located in the zeta chains of the CD3 complex. Due to the intracellular function specificity of Lck, we compared the active sites of both proteins to predict a possible involvement of the pocket shape and the electrostatics in this substrate selection.

The Zernike descriptor approach has also been applied to describe the binding groove features of the major histocompatibility complex (MHC) of class I. In this context, we focused on human leukocyte antigen B*27 (HLA-B*27), which is involved in ankylosing spondylitis (AS), an inflammatory rheumatic disease affecting the axial skeleton.^35,36^ In particular, the subtype HLA-B*2705 is the ancestral allele, which has been found to be associated with AS in almost all investigated populations. Some alleles, such as HLA-B*2709, act as a non-AS-predisposing factor. The HLA-B*2705 and HLA-B*2709 alleles differ by the unique polymorphism at residue 116 (Asp to His): this single substitution is critical for the structural and dynamical features as well as for the T cell repertoire distinguishing the two B27 alleles.^37−39^

Despite the fact that these two HLA-B*27 subtypes manage to bind the same epitope, the 9-residue-long peptide (RPPIFIRRL-pEBNA3A or 9-mer) is recognized by the CD8+ T cell only if presented by HLA-B*2705.^40^ In a recent work, we found that the introduction of a lysine in the N-terminal region of the peptide allows the T cell receptor (TCR)-mediated detection for both subtypes.^40^ Although our previous results have shown a peptide-induced conformational change of the binding groove, a detailed study of its shape and electrostatic behavior is missing.

## Theory

### The Zernike Descriptors

The geometrical and physicochemical properties of the molecule can be represented as three-dimensional functions through the voxelization procedure.^29^ By such an approach, it is possible to summarize the functions as an ordered set of Zernike descriptors.

Given a function , *f*(*r*, θ, ϕ), the Zernike formalism is based on a series expansion in an orthonormal sequence of polynomials
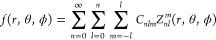
1where *Z*_*nl*_^*m*^ are the 3D Zernike polynomials, and the coefficients *C_nlm_* are the Zernike moments.

Selecting the order *N* at which the sum over *n* is truncated, the level of the approximation is chosen. In this work, we describe the functions representing the molecular shape and surface electrostatic potential using *N* = 20, corresponding to 121 coefficients for each function.

Indeed, it is possible to define the 3D Zernike moments of a function as
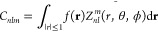
2where *Z̅* is the polynomial complex conjugate.

To obtain the descriptors invariant under translation and rotation, it is necessary to compute the norm (the sum over the index *m*) of the Zernike moments. Therefore, the 3D Zernike descriptors (3DZDs) are defined as
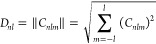
3

A more complete description of the Zernike formalism can be found here.^30,31^

Despite the fact that 3D Zernike descriptors were introduced in the field of structural biology only recently, a significant number of works^33,41−43^ deal with them.

Initially, 3DZD has been applied to compare global protein structures. The rotational invariance of 3DZDs allows a fast comparison between structures as it essentially reduces to a comparison between vectors.^33,41^ Moreover, the geometrical and physicochemical comparison between different proteins can help in identifying common features shared by different protein pockets, thus allowing us to predict possible favorable ligand–receptor pairs.^42^ The application of the Zernike formalism is also well suited in the field of protein–protein docking^43^ as it allows us to quantitatively evaluate the complementarity between protein patches.

Nevertheless, we apply here for the first time a Zernike-based approach to describe the time evolution—as provided by MD simulations—of important protein regions of c-Src kinases and major histocompatibility complexes.

## Methods

### Molecular Dynamics

After sensitivity analysis, our approach has been applied to a set of 400 representative structures, as extracted from previous MD simulations.^40,44,45^ The details of the MD simulations are provided in our recently published works.^40,44,45^ A summary of these simulations is reported in the next two subsections. All the analyses repeated on a duplicate set of MD simulations confirmed our findings (see Figures S4 and S5 in the Supporting Information).

### c-Src and Lck Kinase Domain Simulations

Starting from the crystallographic open structures (PDB IDs: 1y57 and 3lck for c-Src and Lck, respectively), 200 ns-long MDs were run using the Amber99sb force field and the SPC water model. The Verlet cut-off scheme was used and long-range electrostatic interactions were treated by means of the particle-mesh Ewald method. The velocity rescale algorithm was used to keep the temperature constant (300 K). The Gromacs software package version 5.0.5 and version 2016.4 were used for c-Src and Lck simulations, respectively.

### HLA-B*27 Subtype Simulations

We performed molecular dynamics simulations for each HLA-B*27 subtype in complex with the corresponding 9-mer and 10-mer peptides (pEBNA3A with amino acid (a.a.) sequence RPPIFIRRL; pKEBNA3A with a.a. sequence KRPPIFIRRL).^40^ The Gromacs software package version 5.0.7^46^ and the OPLS-AA force field were used.^47^ All the simulations, lasting ∼200 ns each, were performed in a cubic box with the SPC/E water model.^48^ The systems were neutralized and simulated at a physiological concentration of Na^+^ and Cl^–^ (0.15 M). The temperature and pressure were kept constant by means of the velocity rescale algorithm and the Parrinello–Rahman barostat, respectively. The crystallographic structures (PDB codes: 1OGT for B*2705 and 1OF2 for B*2709) were used as starting structures for the MD simulations of the 9-mer and 10-mer complexes.^40^

### Construction and Comparison of Zernike Descriptors

First, we calculated the electrostatic potential by assigning to each atom of the system a partial charge, as obtained using the PDB2PQR algorithm.^49^ For each structure sampled by the MD simulations, we estimated the solvent-accessible surface (SAS) of the chosen set of atoms by defining the protein region to be described by the 3DZD. The regions selected in this work are reported in Table 1.

**Table 1:** List of the Residues Used To Define the Binding Sites

Src binding site	252–257; 275–291; 339–362^34^
Lck binding sites	274–279; 297–313; 384–421^34^
HLA-B*2705(2709) binding sites	5–170^54^
(pEBNA3A or 9-mer) sequence	RPPIFIRRL
(pKEBNA3A or 10-mer) sequence	KRPPIFIRRL

By such an approach, the geometrical and electrostatic properties of the surface generated by the selected set of atoms are described by the 3DZD.

In line with our previous work,^29^ we choose to use 20th-order polynomials, resulting in three 121-dimensional vectors of numbers. A vector describes the shape properties, while the other two describe the positive and negative contributions of the electrostatic potential, respectively. Note that we need to treat separately the two electrostatic contributions since the Zernike formalism does not differentiate positive and negative values but only patterns of nonzero values.^42,50^

Such representation makes it possible to easily compare protein regions even if they differ in terms of orientation and/or number of atoms. To this end, the Manhattan distance has been used as a metric to compare different 3DZDs. Given two vectors **T** and **V** of 121 components, the Manhattan distance between them is defined as
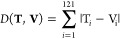
4

Given two patches, A and B, and establishing the Manhattan distance as the metrics between 3DZDs, when we analyze the similarity between protein pockets, we have
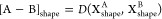
5
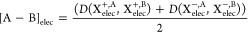
6where X_shape_, X_elec_^+^, and X_elec_^–^ are, respectively, the shape, the electrostatic positive potential, and the electrostatic negative potential 3DZDs. Therefore, the shape similarity is defined as the distance between the shape 3DZDs, and electrostatic similarity is nothing but an average between positive potential similarity and negative potential similarity.

On the other hand, when we study binding complementarity, the electrostatic surface comparison is slightly different because, to achieve high complementarity, the 3DZD describing the positive potential of one patch has to be similar to the negative potential 3DZD of the interacting patch (and vice versa). Therefore, using the same notation than before, the complementarity between the patches A and B is defined as follows
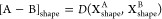
7
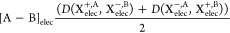
8

Therefore, the shape complementarity between two molecular patches is defined as the distance between their 3DZDs, while the electrostatic complementarity is defined as the cross-average distance between positive and negative potential 3DZDs. Note that both high similarity and high complementarity are achieved when these distances are small.

Given a molecular dynamics trajectory, we calculate the Zernike coefficients for each selected patch at different frames. Therefore, each patch has been described by a set of vectors, and each of these vectors corresponds to a conformational state, as given by molecular dynamics. The comparison between two protein regions is then realized by comparing all the patch conformational states, giving rise to a distribution of Zernike distances.

To compare a pair of Zernike distance distributions, the overlap coefficient has been used.^51^ The overlap represents the fraction of distribution density area of one distribution common to the other. It intuitively follows the value of overlap being between 0 ( when the two distributions are disjoint) and 1 (when the two distributions are identical).

To automatically determine which distributions is characterized by higher values, a sign to the overlap coefficient has been assigned (the sign of overlap is positive/negative if the mean of the first distribution is higher/lower than the mean of the other one). Therefore, given two density functions, *f*_1_(*x*) and *f*_2_(*x*), the overlap coefficient is defined as
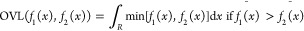
9
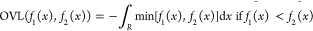
10

The Manhattan distance between two sets of Zernike descriptors and the overlap coefficient between a pair of Zernike distance distributions have been computed using the “dist” and “density” functions of “stats”^52^ R package, respectively.

The calculation of the Zernike coefficients is made using the Python code described in ref (53).

## Results

The purpose of this work is to use the Zernike formalism to provide a quantitative and physically sound description of the protein pockets, allowing us (i) to characterize their time evolution, (ii) to compare different protein pockets, and (iii) to gain insight into the complementarity between pockets and ligands.

In particular, we present the application of the Zernike-based description to characterize the binding sites of two kinases as well as to highlight how the substitution of a single a.a. residue can affect the ligand–receptor complementarity in the major histocompatibility complex (MHC).

### Src and Lck: The Analysis of the Binding Sites

We used the 3DZDs to describe the binding pockets of two kinases (c-Src and Lck), which are very similar in terms of both structure and sequence, at both global and local (pocket) levels. For each frame of the MD trajectories, the shape and electrostatic 3DZDs have been computed using the list of residues defining the binding region (see Table 1).

To study the time evolution of these patches, we analyze the distance between the shape and electrostatic Zernike vector along the MDs and the reference Zernike vector of the starting geometry.

**Figure 1:**
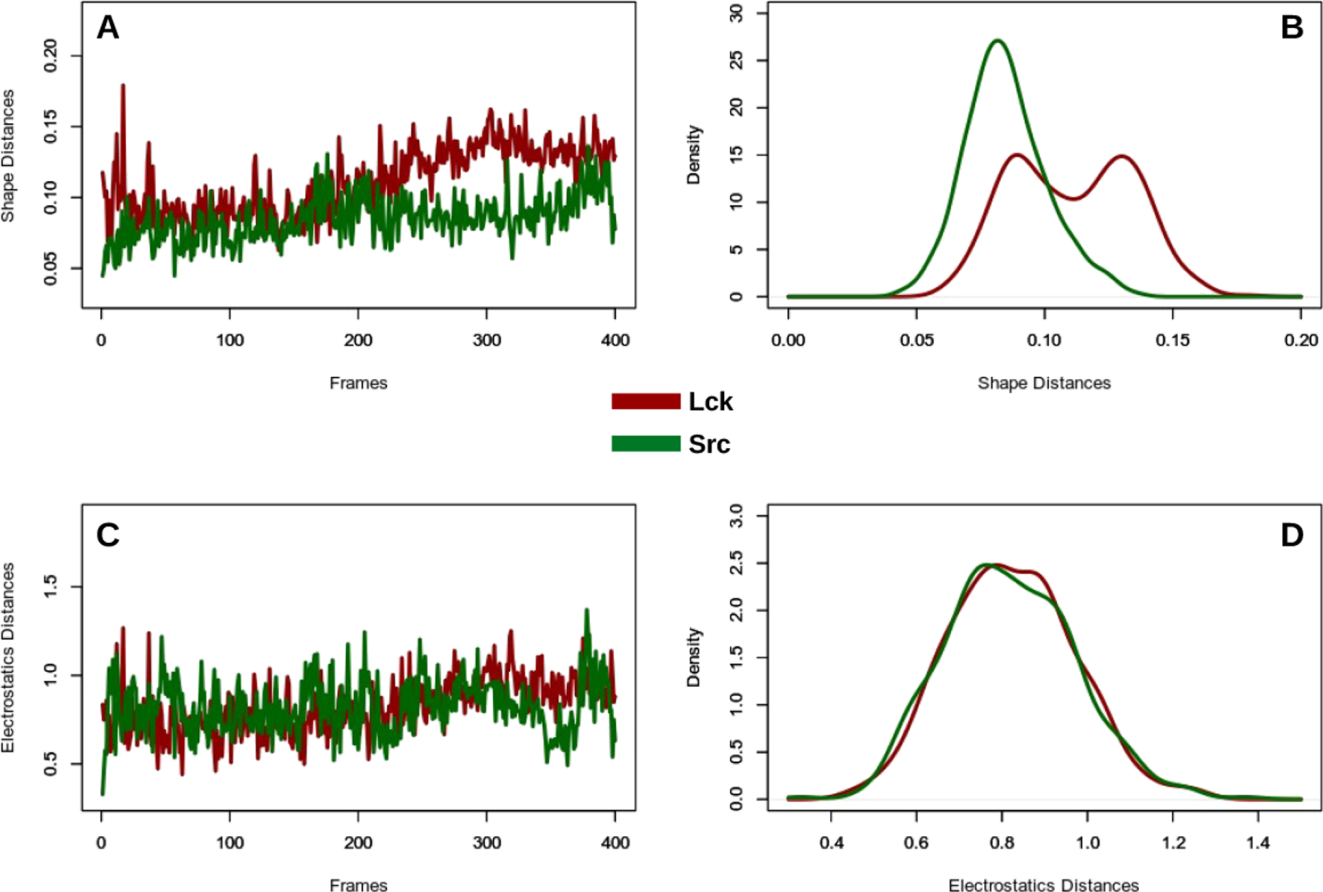
Representations of Zernike along the Lck and Src molecular dynamics simulations; the figures are obtained using distances, in terms of 3DZD, between the Src and Lck binding sites. (A, C) Distances between the binding site of the starting structure and the binding site at the *i*th frame. (B, D) Distributions of the distances as obtained from the MD frames.

We observe that both proteins explore, along the trajectory, a single state, as defined by the electrostatic 3DZD. Interestingly, the Lck binding pocket shows a bimodal distribution of the shape 3DZDs, indicating two slightly different states (Figure 1).

This analysis, in analogy with usual root-mean-square deviation of the atomic positions, provides the equilibrium behavior of local properties, which are supposed to play a major role in the binding behavior.

**Figure 2:**
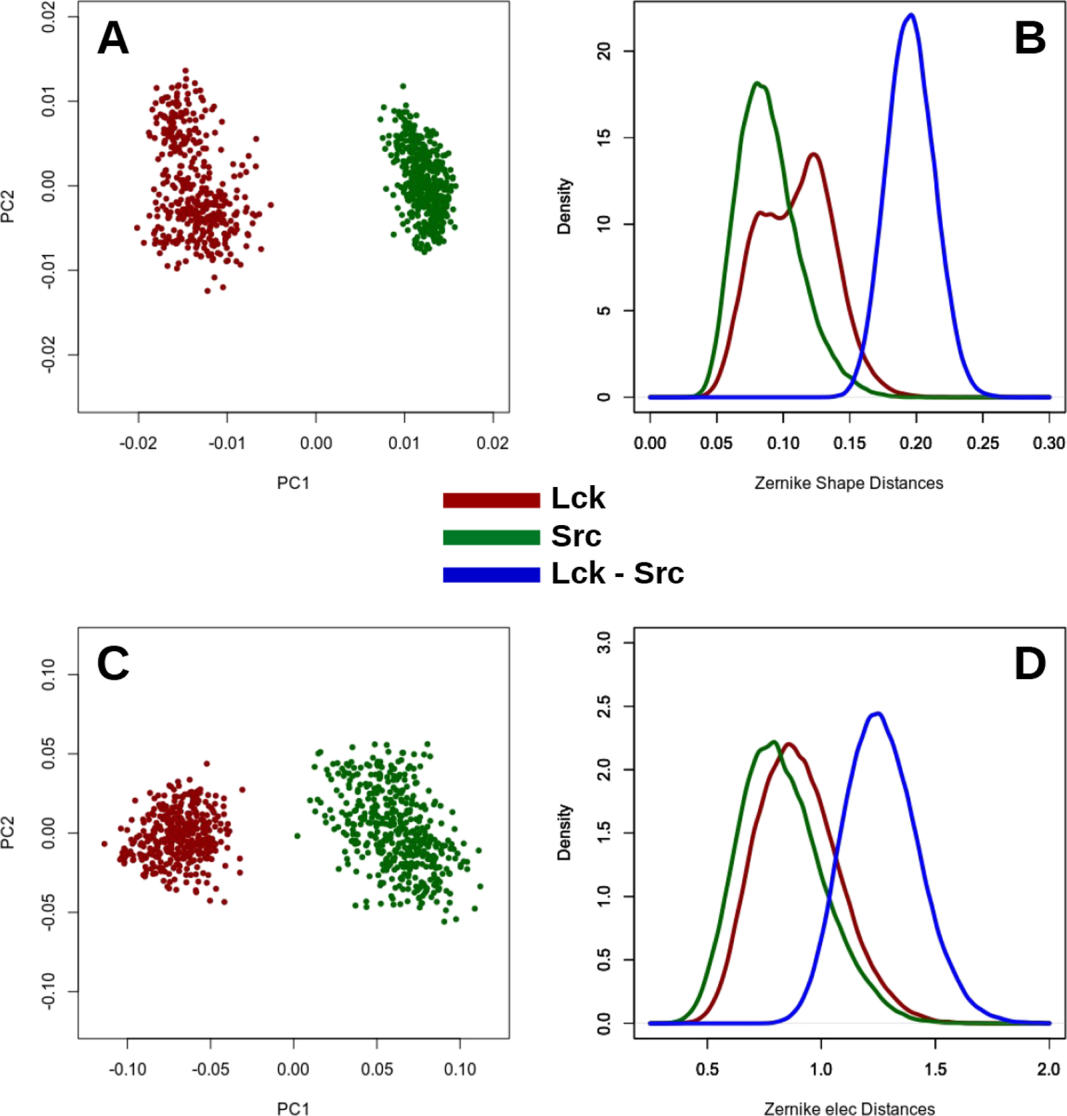
Differences in terms of Zernike descriptors between the Src and Lck binding sites. (A, C) Projections of the Lck and Src 3DZD vectors on the first two principal components. (B, D) Distance distributions of the binding pocket of Src and Lck: D-Intra_Src_, D-Intra_Lck_, and D-Inter_Src-Lck_*.* Top (A, B) and bottom (C, D) panels refer to the shape and electrostatic 3DZDs, respectively.

To better characterize the differences between Src and Lck pockets, we projected each Zernike vector obtained for each MD frame into the essential space defined by the first two eigenvectors as given by principal component analysis (PCA) of the 3DZD vectors. The projection of the 3DZD vectors on the two eigenvectors associated with the largest eigenvalues (describing the 73% of the total variance of the data) clearly shows that the two pockets explore different regions within such a subspace (Figure 2A,C).

A more direct comparison of patch variability in molecular dynamics has been performed by considering the distributions of the distances between the 3DZDs, as obtained by the comparison between all the possible pairs of frames of the three simulations. That is, the 3DZD vector comparison has been performed between Lck and Src as well as within the same proteins. The distance distribution between patches exclusively belonging to Src (Lck) is named D-Intra_Src_ (D-Intra_Lck_). Similarly, the distribution obtained by calculating the distance between each Zernike vector of the Src patch with each Zernike vector of the Lck patch is called D-Inter_Src-Lck_.

As expected, the distances between the Src and Lck pockets (D-Inter_Src-Lck_ distribution) are higher, on average, than those observed within the same protein (D-Intra_Src_ and D-Intra_Lck_ distributions) (Figure 2B,D).

To quantify the difference between two pockets during the simulations, we compare all the distributions by considering their overlap (see Methods), which is defined as the area under the curve that is shared by two distributions (i.e., overlap = 1, 0 indicate identical and completely different distributions, respectively).

The results shown in Table 2 underline the fact that the Zernike distances between different pockets are significantly higher than the distances between the same pockets, as observed along the MD trajectory. On the other hand, the comparison between Intra_Lck_ and Intra_Src_ (or vice versa) shows that the magnitude of the pocket variations in both kinases is analogous.

**Table 2:** Shape and Electrostatic 3DZD Overlaps between the Distributions of the Distances of Src and Lck Binding Pockets^a^

distance		Intra_Src_	Intra_Lck_	Inter_Src-Lck_
shape	Intra_Src_	1	–0.66	–0.02
	Intra_Lck_	0.66	1	–0.05
	Inter_Src-Lck_	0.02	0.05	1
elec	Intra_Src_	1	–0.83	–0.21
	Intra_Lck_	0.83	1	–0.28
	Inter_Src-Lck_	0.21	0.28	1

a These data are obtained from the distributions reported in Figure 2.

Such a result suggests that our approach is able to pinpoint differences between similar protein regions and to provide a quantitative measure of such differences, as provided by the Zernike distance trajectories. It is likely that such differences can play a role in the molecular recognition of specific substrates.

### HLA-B*27 Subtypes and Ligand Peptides: Shape and Electrostatics of Pocket Similarity and Protein–Peptide Complementarity

In this section, we report on the Zernike moment-based method applied to the evaluations of small structural changes as those determined by a single polymorphism. To this end, we focused our analysis on two subtypes of HLA (namely, HLA-B*2709 (HIS 116) and HLA-B*2705 (ASP 116)) bound to two peptides (namely, 9-mer and 10-mer), resulting in four different peptide–HLA complexes.

In particular, we first compared the binding region of the two subtypes bound to the 9-mer peptide by performing the analysis applied to the Src kinases (see previous section).

**Figure 3:**
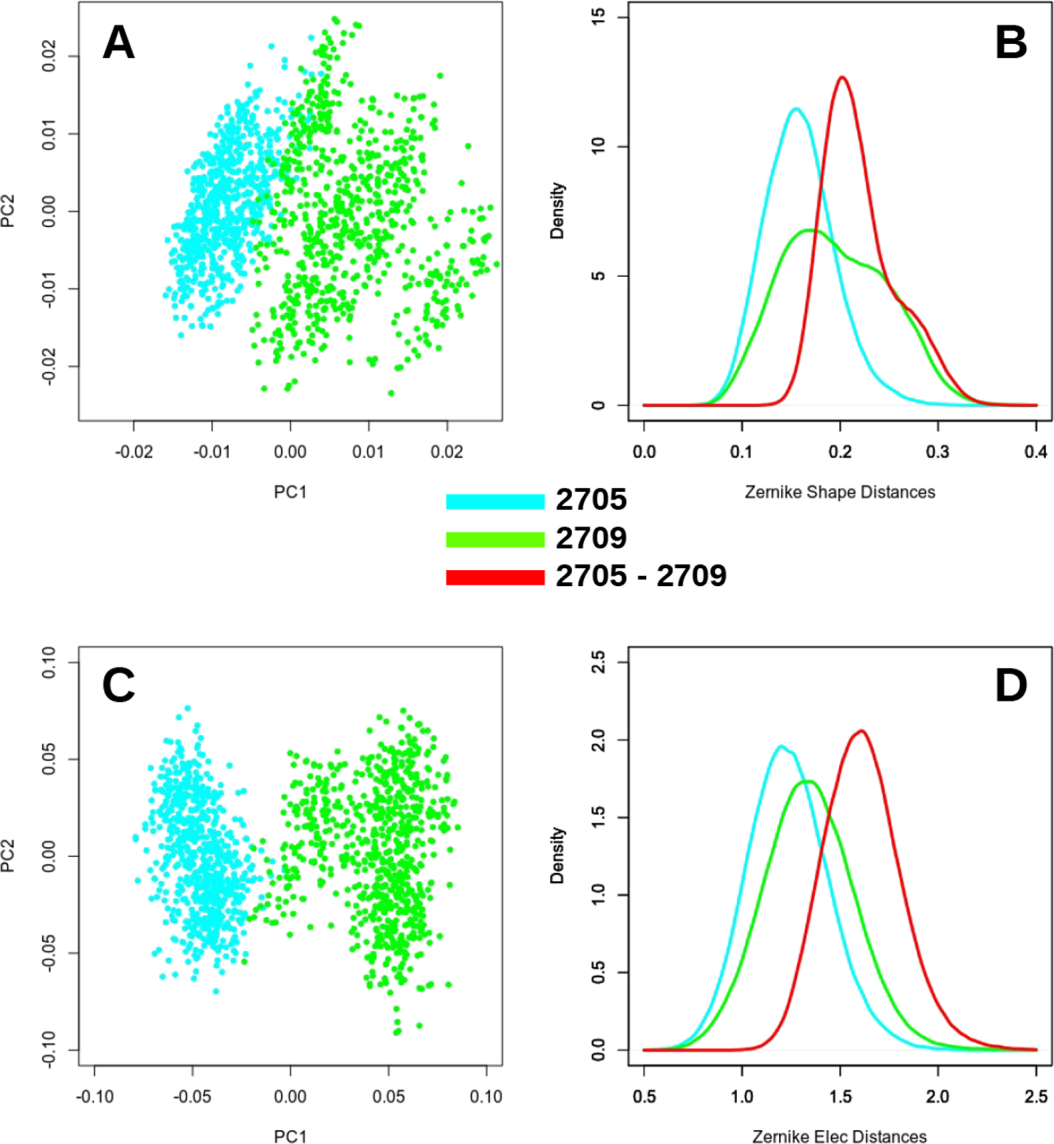
Differences in terms of Zernike descriptors between the HLA-B*2705 and HLA-B*2709 binding sites. (A, C) Projections of the HLA-B*2705 and HLA-B*2709 3DZD vectors on the first two principal components. (B, D) Distance distributions of the binding pocket of HLA-B*2705 and HLA-B*2709: D-Intra_HLA-B*2705_, D-Intra_HLA-B*2709_, and D-Inter_HLA-B*2705-HLA-B*2709_. Top (A, B) and bottom (C, D) panels refer to the shape and electrostatics, respectively.

In a recent published work,^40^ we found that the bound peptide induces some conformational changes in the binding pocket, which provide a different ligand presentation to the T cell receptor. Our results point out that, despite the fact that the differences between these two regions are due to a unique a.a. residue, both the PCA and the distribution distances can capture the differences between the two binding pockets (see Table 3 and Figure 3).

**Table 3:** Shape and Electrostatic Overlaps between the Distribution of the Distances of HLA-B*2705 and HLA-B*2709 Binding Sites as Provided by Molecular Dynamics Simulations^a^

distance		Intra_2705_	Intra_2709_	Inter_2705–2709_
shape	Intra_2705_	1	–0.68	–0.38
	Intra_2709_	0.68	1	–0.69
	Inter_2705–2709_	0.38	0.69	1
elec	Intra_2705_	1	–0.80	–0.35
	Intra_2709_	0.80	1	–0.52
	Inter_2705–2709_	0.35	0.52	1

a These data are extracted from the distributions reported in Figure 3.

As mentioned above, the Zernike description of the molecular surface can be also used to estimate the complementarity between interacting patches.

To this end, we estimate the complementarity between two patches by calculating the difference between their 3DZD vectors. Indeed, since the Zernike polynomials are invariant by rotation and translation, the higher the complementarity between two patches, the lower the distance between their corresponding Zernike vectors (see Methods). On the other side, the electrostatic compatibility (or the complementarity between the two electrostatic potential functions) is achieved when the 3DZDs describing the positive potential of one patch are similar to the negative potential 3DZDs of the other patch.

To quantitatively estimate the interaction behavior between two (part of) molecules, we evaluated the complementarity—as provided by the Zernike descriptors (see Methods)–between the binding regions of HLA-B*27 and the corresponding peptide.

**Figure 4:**
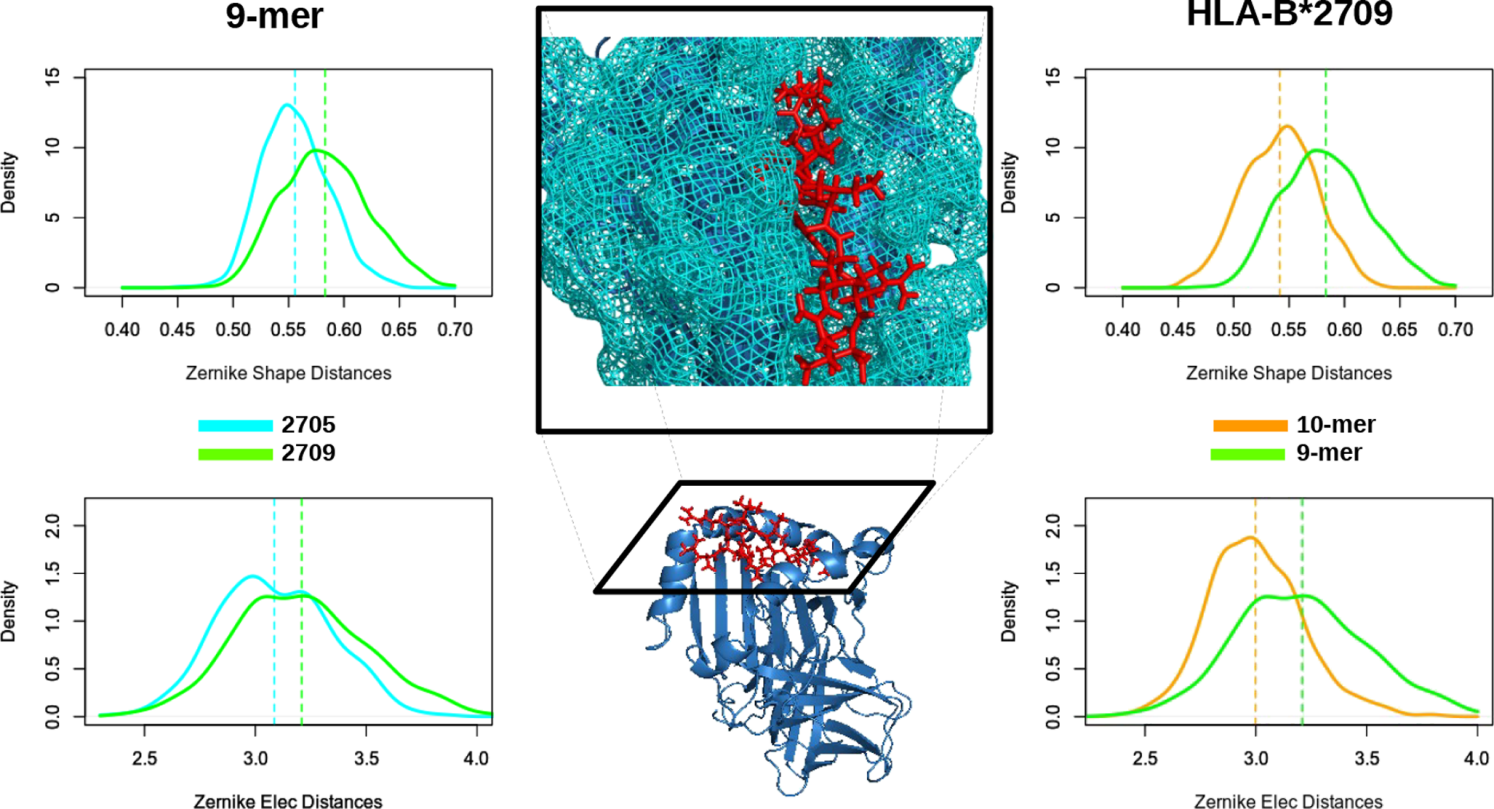
Complementarity distance between HLA and the associated peptides. The dashed lines represent the mean values of distances. Left: HLA-2705 and HLA-2709 bound to 9-mer. Right: HLA-B*2709 bound to 9-mer and 10-mer. Middle: Molecular representations of HLA (blue) bound to 9-mer (red).

Interestingly, our data report that HLA-B*2705 shows a higher complementarity with the 9-mer peptide than HLA-B*2709, as shown in Figure 4 and quantified by the overlap (see Table 4). Indeed, both in terms of shape and electrostatics, the complementarity is higher when 9-mer interacts with B*2705 than when 9-mer interacts with B*2709. Then, we studied the difference in complementarity when HLA-B*2709 is in complex with 9-mer or 10-mer, and both the shape and electrostatic 3DZDs detect a higher complementarity when 10-mer is bound with the analyzed HLA-B*27 subtypes (Figure 4).

**Table 4:** Shape and Electrostatic 3DZD Overlaps between HLA-B*2705/9-mer and HLA-B*2709/9-mer (First Two Rows) and HLA-B*2709/9-mer and HLA-B*2709/10-mer (Last Two Rows)^a^

complementarity distance		2705–2709
9-mer	shape	–0.70
	electrostatics	–0.84

complementarity distance		10-mer–9-mer
HLA-B*2709	shape	–0.59
	electrostatics	–0.67

a The associated distributions are shown in Figure 4.

We finally compared the complementarity of 10-mer with the two HLA-B*27 subtypes. Interestingly, the shape 3DZD assigns a very similar complementarity value to these two molecular complexes, in line with experimental activity,^40^ even if the electrostatic 3DZD description highlights a preference in binding between 10-mer and HLA-B*2709 with respect to HLA-B*2709 (see the Supporting Information).

## Conclusions

In this work, we applied the Zernike formalism on molecular dynamics data to represent the properties of specific molecular regions in a very compact form.

Once the residues defining the region of interest are identified, the overall shape and electrostatic characteristics are summarized in 121 ordered numbers, that is, the norm of the coefficients of the Zernike expansion. Such a compact description of the molecular patches consent to easily calculate the distance between any possible pair of vectors (corresponding to different surfaces) and their behavior along an MD trajectory.

This method, not requiring any preliminary structural superposition, provides a description, which does not depend on the dimension of the region of the molecule described; that is, it allows us to compare regions of different sizes in terms of number of residues and residue type.

We showed that 3DZD can be used to detect shape similarity as well as to analyze the complementarity between interacting molecular partners. We investigate how the structural–dynamical evolution of the systems modifies the shape and the electrostatic properties of a protein region, eventually affecting the binding with its molecular partner.

Moreover, the application of the Zernike formalism to an extended region of the conformational space provides statistically significant results, thus increasing its reliability and robustness with respect to single-structure calculations.

The application of the method to different biologically relevant systems shows that is possible to identify differences even between very similar pockets, such as in the cases of the HLA-B*2705 versus HLA-B*2709 and Src versus Lck. Our data suggest that the shape complementarity—more than the electrostatics—could contribute in determining a diverse presentation of the epitope to the TCR. For all the proteins in the study, the shape complementarity seems to play a major role in characterizing the molecular interaction behavior in these systems.

## Supporting Information Available

Further analysis about the application of the Zernike formalism to molecular dynamics data (PDF)
