# Supplementary material for: Quantitative Characterization of Binding Pockets and Binding Complementarity by Means of Zernike Descriptors
Source: J Chem Inf Model. 2020 Feb 12;60(3):1390–8. doi: 10.1021/acs.jcim.9b01066 (PMC7997106; doi:10.1021/acs.jcim.9b01066)
Supplement: Supplementary file 1 — ci9b01066_si_001.pdf [file ci9b01066_si_001.pdf]

Supporting Information: Quantitative Characterization of Binding Pockets and Binding Complementarity by means of Zernike Descriptors

Lorenzo Di Rienzo,[†] Edoardo Milanetti,[†,‡] Josephine Alba,[¶] and Marco D'Abramo[*,¶]

†*Department of Physics, Sapienza University of Rome, Piazzale Aldo Moro, 5, 00185 Rome, Italy.*

‡*Center for Life Nano Science@Sapienza, Italian Institute of Technology, Viale Regina Elena 291, 00161 Rome, Italy.*

¶*Department of Chemistry, Sapienza University of Rome, Piazzale Aldo Moro, 5, 00185 Rome, Italy.*

E-mail: *marco.dabramo@uniroma1.it

Sensitivity analysis of the pocket definition

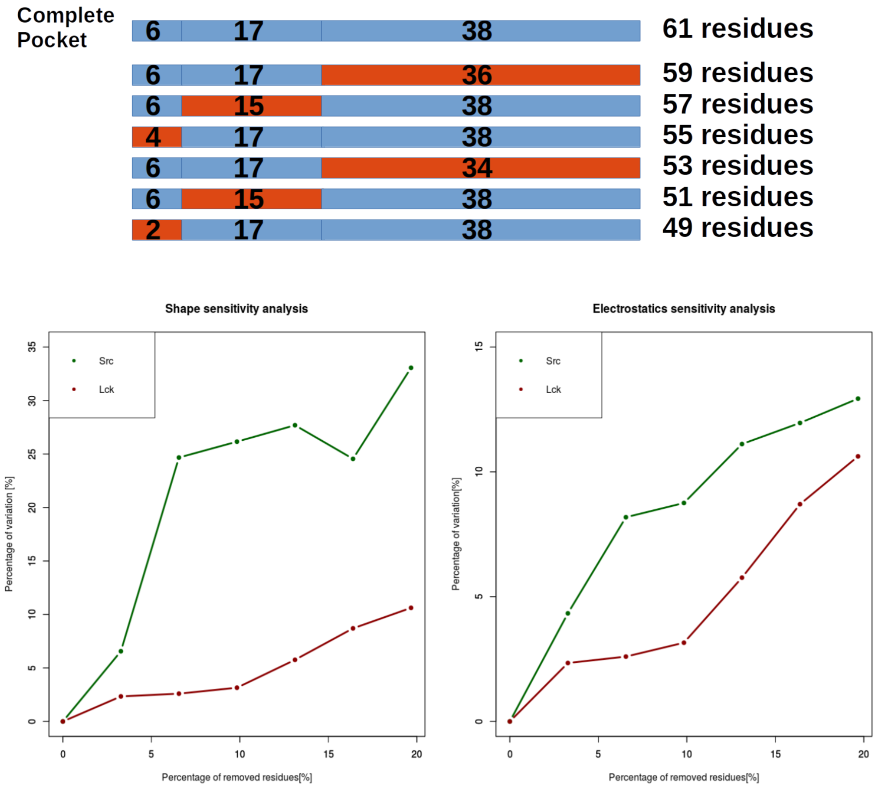

Figure S1: A method sensitivity analysis in regard to the definition of the residues list composing the Kinases binding sites. In the upper part we illustrate the changes in the pocket definition, where we progressively remove two terminal residues from each stretch of the binding groove. In the bottom plots we report the normalized manhattan distance between the 3DZD of the original binding site and the 3DZD of the reduced ones, both in terms of shape and electrostatics.

To better understand how the method is sensible to the definition of the residue list, a sensitivity analysis has been performed on the case of the kinases. The three contiguous stretches of residues defining the pocket have been changed by progressively removing two terminal residues from each stretch (see Figure S1). The changes due to the residue deletion - expressed as the manhattan distance between the 3DZD of the original binding site and the 3DZD of the reduced one - has been reported versus the extent of the residue list variation. In order to give a clear understanding, the magnitude in y-axis of the plots are normalized to the mean distance experienced by the pocket in molecular dynamics, in terms of 3dzd.

Therefore we analyzed the variation in 3DZD caused by slightly different definition of binding sites with respect to the mean variation in molecular dynamics.

Such a figure points out that small changes in pocket definition cause a variability in 3DZD that are much smaller than the typical variability experienced , because of the thermal noise, during a time evolution. In the worst case, when 12 residues (out of 61) were removed from the Src binding site, the (shape) mean distance with respect to the original binding pocket is the 34% of the mean distance obtained between the different frames using the original definition of binding pocket. These results make us confident that our approach is quite unaffected by limited variation in the cavity definition.

Cartesian Coordinates analysis of the pockets

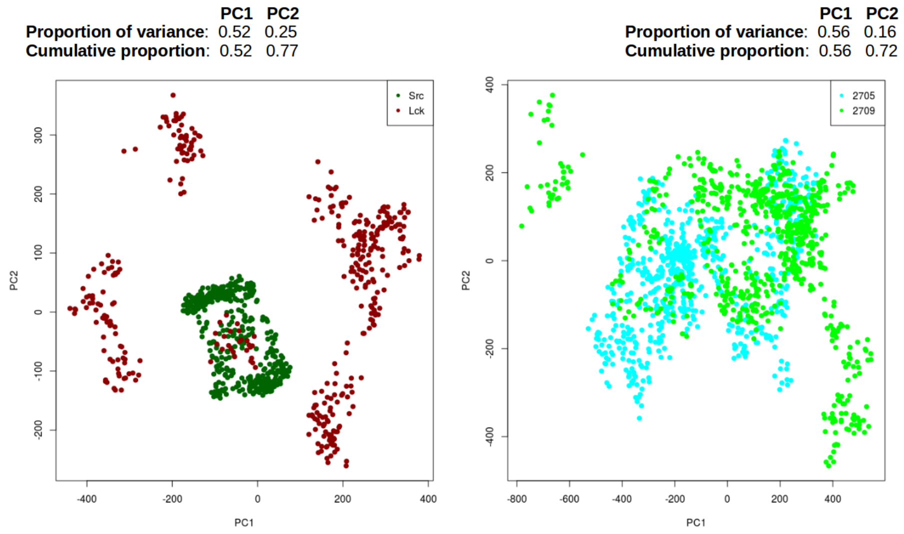

Figure S2: PCA analysis of the pocket regions. Left panel: Src-Lck, right panel: HLA 2705-2709.

The possibility to discriminate between different molecular patches using the cartesian coordinates has been analyzed by means of Principal Component Analysis (PCA)(Figure S2). The projection of the coordinates of the alpha carbon atoms on the principal subspace shows that Lck cavity fluctuates more then the Src counterparts. Such a higher shape variability (also evident from the panels A-B of figure 2) precludes the possibility to distinguish between Src and Lck cavity.

A similar behavior is also found in the case of the HLA (Figure S2 - right panel). These results point out that the coordinates-based analysis might not detect the subtle differences between similar structural regions. Finally, it is also worth mentioning that the Zernike approach also characterizes the electrostatics of the systems, which is assumed to give an important contribution to the binding processes.

Analysis of the overlap convergence

To test the convergence of the observed overlaps, all the molecular dynamics trajectories have been divided in 3 parts. Then, we calculated the overlaps between the distributions of these subpartes, resulting in 9 overlap values which have been used to estimate the statistical error. The results, reported in Table S1, clearly show our estimate of the statistical error is quite small, thus assuring that the overlap achieves a reasonable degree of convergence within our simulation time.

Table S1: Overlap values with their statistical errors.

	**Distributions**	**OVL**	σ(**OVL**)
**Shape**	Intra-Lck — Intra-Src	0.66	0.13
	Intra-Lck — Inter-LckSrc	0.05	0.01
	Intra-Src — Inter-LckSrc	0.02	0.01
**Elec**	Intra-Lck — Intra-Src	0.83	0.04
	Intra-Lck — Inter-LckSrc	0.28	0.05
	Intra-Src — Inter-LckSrc	0.21	0.04
**Shape**	Intra-05 — Intra-09	0.68	0.11
	Intra-05 — Inter-0509	0.38	0.08
	Intra-09 — Inter-0509	0.69	0.05
**Elec**	Intra-05 — Intra-09	0.80	0.04
	Intra-05 — Inter-0509	0.35	0.06
	Intra-09 — Inter-0509	0.52	0.11

Further complementarity analysis of HLA and ligands

Table S2: The overlap, both for shape and electrostatic 3DZD, between the distribution of complementarity distances of 10-mer and the 2 HLA mutants in molecular dynamics. This table refers to the distributions reported in Figure S3

Complementarity distances		(2705 - 2709)
**10-mer**	Shape	- 0.87
	Electrostatics	0.43

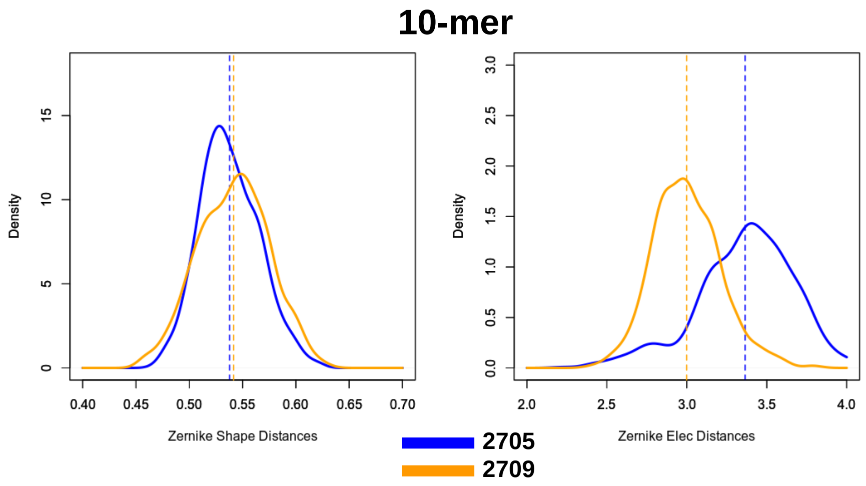

Figure S3: Complementarity distance of the interaction between 10-mer and the HLA mutants. The left figure refers to the shape formalism while the right figure regards electrostatics. The complementarity is achieved when are recorded low distances between molecular patches.

Analysis of duplicated simulations

We duplicate all the molecular dynamics simulations of the HLA-peptides complexes.

As in the reference simulations, for each frame of the corresponding trajectories we re-calculated the shape and the electrostatics 3DZD for both the HLA binding groove and the cognate peptides.

As shown in figure S4, the principal component analysis on the shape and electrostatic is able to discriminate between the pockets of the HLA 2709 and the 2705 mutants, thus confirming the reliability of our results.

Concerning the shape and electrostatic complementary analysis (Fig. S5), we report as a representative example the complementary distributions of the 9-mer for the 2705 and 2709 systems as obtained in the duplicate simulations. Also in this case, the results confirm the previous findings.

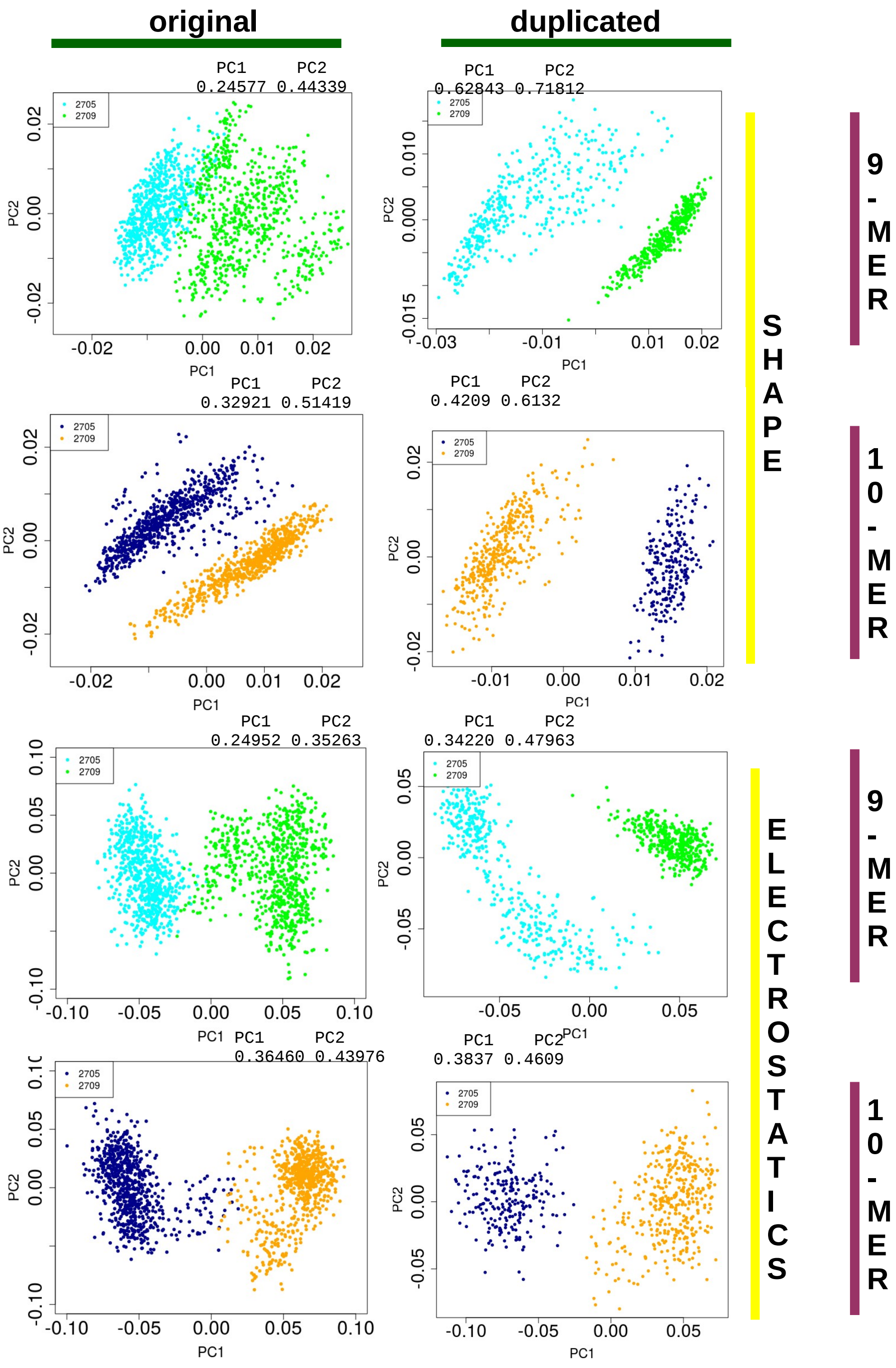

Figure S4: Comparison of the shape and the electrostatics 3DZD PCA analysis applied to two independent sets (left and right panels, respectively) of MD simulations.

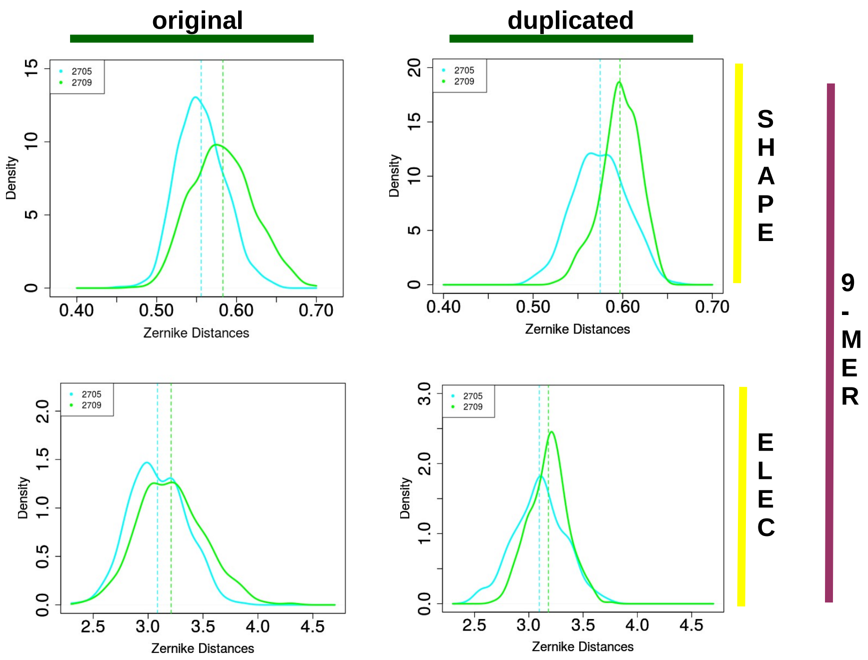

Figure S5: Comparison of the shape and electrostatic complementarity as obtained from two set (left and right panels, respectively) of MD simulations.
